# Exploring the impact of total quality management initiatives on construction industry projects in Pakistan

**DOI:** 10.1371/journal.pone.0274827

**Published:** 2022-09-27

**Authors:** Nimra Afzal, Aamer Hanif, Muhammad Rafique

**Affiliations:** 1 School of Management, Air University, Islamabad, Pakistan; 2 Islamabad Business School, Islamabad, Pakistan; University of Salento, ITALY

## Abstract

The impact of total quality management on organizational performance has been studied extensively, however, the impact of total quality management initiatives on project performance is an area of ongoing research. The key objective of this research is to explore the impact of total quality management initiatives on project performance in the construction industry of Pakistan. Data was collected from 326 personnel working at different management levels across some of the leading construction firms operating in Pakistan. Analysis revealed that operational focus, management commitment, and employee involvement were deemed as dominant total quality management factors affecting project performance in the construction industry. Mediation analysis revealed a significant relationship between employee involvement and project performance mediated by management commitment. Research limitations and directions for future research have also been identified.

## 1. Introduction

In recent decades, using "total quality management" (TQM) initiatives has been a key stratey for achieving consumer satisfaction and for enhancing organizational performance. Total quality management is considered an approach for managing and continuously improving the entire organization to enhance efficiency of a business through active participation of every organizational member. The fundamental concept of total quality management was introduced in 1940 by three gurus; Deming, Juran and Feigenbaum in Japan who used the term "total quality" during the first international conference on quality control held in Tokyo [1]. In the 1980s and 1990s, the new quality control and management phases finally started which got widely known as total quality management.

The first implementation of TQM concept was in Japan’s general manufacturing and automobiles industry. Therefore, most of the literature addresses the industry’s misleading impression that the total quality management concept cannot be implemented in any industry other than the manufacturing industry. One key element of TQM approach is to accomplish customer satisfaction which is an essential goal for any organization including construction firms also where research studies have been conducted in the past [2]. Some of the past researches include TQM implementation in the Palestinian construction industry [3], TQM implementation in the Oman construction industry [2, 4], and a study of the association between TQM and project performance in the Malaysian construction industry [5]. However, the studies mentioned above are limited to country characteristics different from Pakistan which is currently facing economic challenges even though construction related practices are quite similar.

### 1.1 Research gap

Many research studies have been conducted in Pakistan to analyze the influence of TQM initiatives across different performance dimensions, including firm supply performance [6], export performance [7], innovation performance [8], corporate green performance [9], organizational performance [10], business performance [11] and also in public sector universities [12]. While TQM has been given limited consideration in local construction industry, the research focus was on specifics like structural failure, on construction firms having ISO certifications already and on using ICT for modeling [13–15]. Hence, there is a research gap in existing TQM literature in the context of project performance in the Pakistani construction industry. With current focus on improvement of national economy by engaging the construction sector in Pakistan, TQM implementation has the potential to improve project performance and deliver quality outcomes which contribute to the uplift of this sector.

### 1.2 Research objectives

This paper aims to examine the association between TQM and project performance in the context of the construction industry of Pakistan. More specifically, the objectives of this research are as follows:

- To identify and examine the effect of TQM principles on project performance.
- To determine which total quality management principles have significant impact on project performance.
- To examine the mediating role of management commitment between employee involvement and project performance.

The significant contribution of this research is identification of TQM initiatives relevant to the Pakistani construction industry which enable organizational performance and project success.

## 2. Literature review

TQM is a management approach that emphasizes customer satisfaction and continuous improvement in the organization [16]. This is enabled by each employee within the firm who must consider the requirements of the person who uses their output. The objectives of total quality management are to develop quality enhancement as a dominant priority of an enterprise and organizational effectiveness improvements [17]. Arditi et al. [18] defined quality as meeting the expectations of owners, regulatory agencies, designers and builders in the construction industry. TQM is an efficient system that incorporates quality improvement, quality maintenance and quality development to enable service at an economic level for achieving complete satisfaction of customers and clients [19].

PMI clearly explains a project as "a temporary endeavor undertaken to create a unique product or service". The projects are distinctive, have specific goals and objectives, and have a clear starting and ending date. The unpredictable and complex nature of the project causes sensitive and serious challenges to project-based firms. In the context of construction projects, success might be measured differently by construction firms depending on their objectives and goals [20]. Alzahrani & Emsley [21] implied that what is considered a successful measure on one project might well be considered unsuccessful or failure on another. Therefore, project’s success may be determined differently according to the objectives or criteria set by the construction firms or organizations [20]. Hence there is no specific framework for project performance measurement in this industry [22]. It is impossible to establish a specific criteria or standard checklist for measuring project success because of varying characteristics and objectives of projects in terms of location, complexity, uniqueness and size. Project performance indicators include those related to cost, time and quality, also known as the iron triangle; these indicators are commonly accepted to measure construction project success as well [23].

The synthesis of principles and philosophies of TQM researchers in construction-related studies has yielded seven elements of TQM. These elements are continuous improvement, commitment, customer focus, strategic planning, operation focus, employee involvement, measurement, analysis & knowledge management. Few research studies have been conducted on total quality management in the construction industry, for example, Mir & Pinnington, Jong et al. and Leong et al. [5, 24, 25]. These studies found a positive association between total quality management and project performance. Jong et al. [5] explored the link between TQM and project performance in Malaysia. It was observed that total quality management (TQM) significantly affects the performance. Moreover, workforce involvement and continuous operational focus were essential elements for the project’s performance. Leong et al. [25] reported "ISO 9000" certification effectiveness in firms in Malaysia using indicators of project performance. They found that time variance and satisfaction of customers were positively significant with "ISO 9000 certification". No fixed elements of ISO 9000 certification in this research were inspected as indicators of project performance. Mir & Pinnington [24] examined the link between TQM and project success in UAE. The framework project management perfromance assessment (PMPA) of TQM was implemented and examined against the project’s success and it was found that the variables of PMPA had a positive impact on the success of the project.

### 2.1 Commitment and project performance

The effectiveness of TQM system mostly relies on top management commitment and their dedication to organization’s goals and objectives [26, 27]. Top management or executives act as key drivers of the total quality management (TQM) program because they establish goals, systems and values to achieve customer satisfaction [28]. Commitment is essential not only for discussing or achieving business goals, strategies and objectives but also for providing motivation and direction to the workforce of an organization [29]. The successful completion of any work targeted at changing the organizational operations philosophy is robustly connected with upper-level management commitment. Othman et al. [30] argued that the consistent involvement of top management in quality-related activities would facilitate the changing attitudes of employees toward quality in an organization. According to the evidence mentioned above, we can say that commitment positively influences project performance. Therefore, we propose the following hypothesis.

H1: Commitment will have a significant positive effect on project performance.

### 2.2 Employee involvement and project performance

Involvement of employees is about active participation of organizational members in various levels of the decision-making process. Involvement also refers to the sense of commitment and responsibility [31]. Employees at all levels are a vital asset in an organization without which it would not achieve its goals and objectives [32]. Amah & Ahiauzu [33] studied employee involvement and organization’s effectiveness. They found that employee involvement positively influenced the effectiveness of an organization. Bakotić & Rogošić [34] researched employee involvement as the key element of quality practices. Results showed that employee involvement positively affected the implementation of the system management method, process method, continual improvement, and decision-making method. Hence, we propose the following hypothesis.

H2: Employee involvement will have a significant effect on project performance.

### 2.3 Client or customer focus and project performance

TQM is targeted towards a customer-oriented approach. Knowing and understanding the customers and client’s necessities, being responsive to the demands of the client, and additionally, ensuring satisfaction of the customer have led to growth in revenue, profitability, cash flow and market share [35]. Pambreni et al. [36] argued that focus on customers was an essential principle for the success of an organization because it was a starting point in any quality initiative. They studied TQM implementation in food companies and found that customer focus had a significant positive effect on organizational performance in the service sector of Spain. This study also suggests that focusing on clients/customers leads to a better understanding of clients’/customers’ requirements, client/customer satisfaction and improved organizational performance. Zou et al. [37] found that management strategy for customer relationships led to better project performance. Based upon these findings regarding impact of customer satisfaction on project performance, the following hypothesis is suggested.

H3: Client and/or Customer focus will have a significant effect on project performance.

### 2.4 Continuous improvement and project performance

TQM is being termed “a journey, not a destination” [38]. It is about adopting an improvement-centered culture, understanding the customer requirements, and improving the processes to satisfy customers [36]. Continuous improvement’s fundamental idea is to prevent mistakes and defects from recurring [26]. Lizarelli et al. [39] analyzed the association between innovation performance and continuous improvement in the manufacturing industry of Brazil. They found that continuous improvement (CI) had a positive connection with innovation performance. Since CI aims to prevent defects, reduce waste and enhance performance, we propose the following hypothesis.

H4: Continuous improvement will have a significant effect on project performance.

### 2.5 Strategic planning and project performance

Planning is a significant element of the success of the project. In any project, better planning increases the chances of uneventful project execution and enables completion in time [40, 41]. High-quality planning reduces cost and schedule overruns in engineering and construction organizations [42]. Some other factors have also been identified in the literature on project management regarding the significance of planning [43]. These relate to pitfalls of the traditional approach of planning which contains exorbitant control restrictions and reduced opportunities for innovation or creativity eventually leading to project failure. The basic idea behind strategic planning is to try to reduce ambiguity and enhance the chance of success in a project. Although “strategic planning does not guarantee successful project completion; lack of planning most likely leads to project failure” according to PMBOK. Based on the evidence mentioned above, we propose the following hypothesis.

H5: Strategic planning will have a significant effect on project performance.

### 2.6 Operation focus and project performance

Operations management uses methods in which all organizational resources are used in a productive and well-organized way to accomplish goals and desired performance [44]. The focus is on operational activities including proactive and preventive approaches to managing quality [45]. Activities include a stable schedule of production, reducing variation and distribution of work to enhance product quality during the production stage [46]. Different researchers, Irfan & Kee, Mehralian et al., Valmohammadi & Roshanzamir [47–49] conducted studies examining the association between operations, process management and performance. These studies found a significant and positive relationship among these factors. Hence, we propose the following hypothesis.

H6: Operation focus will have a significant effect on project performance.

### 2.7 Measurement, analysis, knowledge management and project performance

The availability of consistent, high-quality, adequate and timely information ensures performance improvement [50]. Bouranta et al. [46] researched the validity and reliability of data and information using measurement and analysis tools to support decisions on quality related issues to improve organizational performance. The collection of data, application of quality tools, analysis procedures and dissemination of useful knowledge enhances the performances of firms [47, 49]. Research conducted by Zeng et al. [51] on impact of soft and hard quality initiatives on innovation performance detected that quality-related data and analysis directly affects project performance. Hence, the following hypothesis is proposed.

H7: Measurement, analysis, and knowledge management will have a significant effect on project performance.

**Fig 1:**
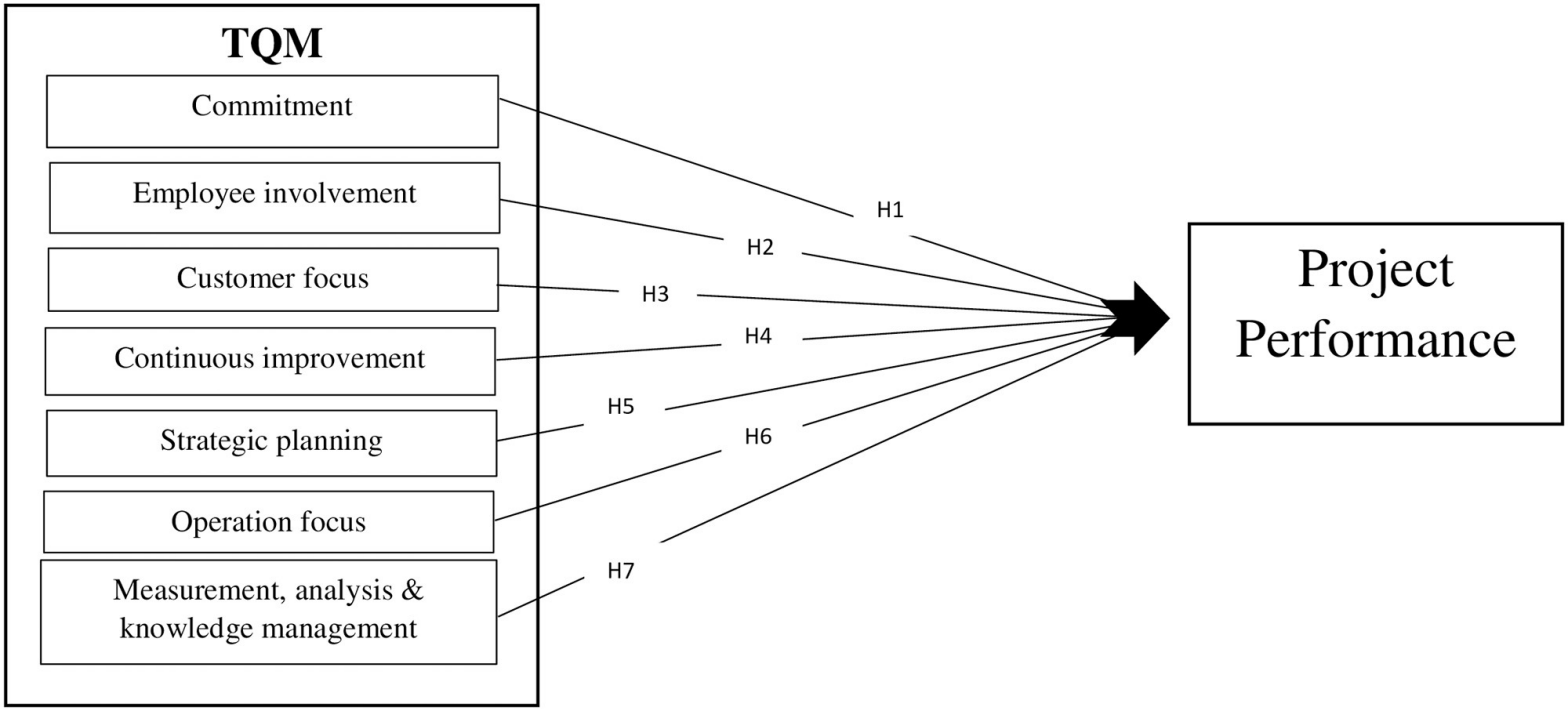
Research model.

Based on literature review presented above and ensuing creation of the research hypotheses, the proposed research model is shown in Fig 1. In this model, clients of the organizations have been generally addressed as customers.

### 2.8 Mediating role of management commitment

**Fig 2:**
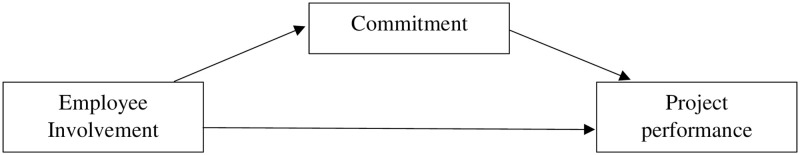
Mediation model.

The impace of management commitment on project performance has been discussed previously. Additionally, this research explores the mediating role of top-level management commitment, support and their involvement in quality-related activities to enhance project performance. Lee et al. [52] researched employees’ attitudes in response to HR development efforts with the moderating role of top management support and found that it moderated the relationship between employees’ attitudes and HRD efforts. Hence, management support for employees’ professional development is an essential factor for the growth and development of an organization because the top-level management members identify and guide the strategic business directions [53]. Another research conducted by Gözükara et al. [54] on the linkage between total quality management and culture development in Istanbul with mediating effect of employee empowerment and management commitment found that commitment mediated the relationship but employee empowerment did not. Commitment is observed as starting point in the direction of implementation and performance of total quality management within an organization [55]. It is impossible to adopt total quality management and achieve desired project performance without the strong support from top management. Moreover, the involvement of employees significantly influences the commitment of employees to an organization [56]. Employee involvement affects the performance of an organization in two ways. Firstly, it increases employees’ productivity, and secondly, it increases the firm’s capacity to react fast to agile decisions and plans made by top management. Hence, employee involvement is genuinely affected by the top-level management support which is a vital aspect of project’s success [57]. The corresponding mediation model based upon this discussion is shown in Fig 2, and subsequently the following hypothesis is proposed.

H8: Commitment will mediate the relationship between employee involvement and project performance.

## 3. Methodology

### 3.1 Data collection and sample characteristics

This study’s participants were employees working in construction firms across major cities of Pakistan. The questionnaire used to collect data is discussed in the next section. Convenience sampling technique was used for data collection. 600 forms were distributed out of which 330 were received back with a response rate of 54.34%. However, 4 were discarded as unusable, leaving 326 responses comprising the final sample size of this study.

With regards to gender distribution, out of the 326 respondents, 92% were males while 8% were female. This was expected as construction industry employs fewer females in Pakistan. Regarding employee job roles and management positions, 32% of respondents were in a lower-level position, 43% respondents were in a middle-level position and 25% of respondents were in a top-level position. About 70% of respondents had a total job experience of up to 5 years while the remaining had experience over 5 years.

### 3.2 Ethical considerations of research

The researchers gave the autonomy of individual respondents for this research due consideration, and all participants in the survey were voluntary. Confidentiality of participants, informed consent, and voluntary participation was specifically ensured. All participants were informed that their identity and individual response were to be treated as anonymous and would be utilized only for this research.

### 3.3 Research instrument and measures

The research instrument had statements requiring responses on five-point Likert scale to measure the constructs (1: strongly disagree, 2: disagree, 3: neutral, 4: agree, 5: strongly disagree). A brief operational definition of research variables and corresponding number of scale items for measurement is given in Table 1. *Commitment* was measured by using 8 item scale [28, 58, 59]. One sample item of this scale is “Organization’s top management has objectives for quality performance”. *Employee involvement* was measured by using a 10-item scale [28, 60]. One sample item of this scale is “Employees are recognized for superior quality improvement”. C*ustomer focus* was measured by using 7 item scale [58, 59]. One sample item of this scale is “The organization frequently is in close contact with its customers”. *Continuous improvement* was measured by using 9 item scale [58]. One sample item of this scale is “The organization has a quality improvement program”. *Strategic planning* was measured by using 5 item scale [61]. One sample item of this scale is "Our organization has a comprehensive, structured planning process which regularly sets and reviews short and long-term goals". *Operation focus* was measured using 7 item scale [59, 61]. One sample item of this scale is "Our organization practices daily operation work processes report system". *Measurement*, *analysis & knowledge management* was measured using 7 item scale [5, 61]. One sample item of this scale is “Our organization implements organizational performance measurement system”. *Project performance* was measured by using 4 item scale [62]. One sample item of this scale is “The project was successful in terms of timeliness of project completion”.

**Table 1:** Operational definitions and number of items.

Variable Name	Definition	Items
Commitment	Top management commitment means the direct participation of high-level officials in an organization’s critical and specific aspects.	8
Employee involvement	Employee involvement in TQM is defined as the process of authorizing employees of an organization to solve problems and make decisions related to quality.	10
Customer focus	To understand what your client needs or wants and figure out the right people, process, and material to meet desired requirements.	7
Continuous improvement	The ongoing improvements in processes, products, and services.	9
Strategic planning	Strategic planning prioritizes the efforts and resource planning for better plan execution.	5
Operation focus	Use resources effectively and efficiently in the production phase.	7
Measurement, analysis, and knowledge management	The availability of consistent, high-quality, adequate & timely information ensures by the organization for all the users for performance improvement.	7
Project performance	Managing projects successfully so that they contribute to organizational performance and strategy. It is about project completion on time, within budget and achieving desired goals.	4

Table 2 shows Cronbach Alpha valules for scale reliability, skewness and kurtosis values for checking data normality and VIF values for determining multicollinearity issues in the data.

**Table 2:** Cronbach alpha and normality test summary.

Variables	Reliability	Skewness	Kurtosis	VIF
Commitment	.795	.226	1.281	2.259
Employee involvement	.803	.493	1.315	2.743
Customer focus	.775	.134	1.945	3.061
Continuous improvement	.780	.824	2.510	2.980
Strategic planning	.700	.755	2.115	2.829
Operation focus	.713	1.029	2.520	1.677
Measurement, analysis & KM	.772	.607	2.741	3.573
Project performance	.885	.341	-.194	

Table 3 shows the mean and SD values of each variable. It also shows the values of Pearson’s correlation coefficient between each variable of the study.

**Table 3:** Descriptive and correlations.

Variables	Mean	SD	1	2	3	4	5	6	7	8
Commitment	3.460	.503	1							
Employee involvement	3.336	.482	.744**	1						
Customer focus	3.397	.521	.569**	.791**	1					
Continuous improvement	3.407	.458	.694**	.774**	.682**	1				
Strategic planning	3.478	.454	.640**	.626**	.616**	.766**	1			
Operation focus	3.456	.447	.519**	.631**	.663**	.644**	.748**	1		
Measurement, analysis & KM	3.426	.505	.522**	.743**	.766**	.660**	.648**	.780**	1	
Project performance	3.427	.699	.492**	.472**	.373**	.452**	.402**	.399**	.370**	1

Note: p< .05*, p< .01**.

The next section is the results where we will report data analysis outcomes and findings.

## 4. Results

SPSS software was used to analyze the collected data. Correlation analysis suggested that associations between variables were significant and in anticipated directions thereby providing introductory support for the research hypotheses. Data normality assessment is a prerequisite for regression analysis and numerous other statistical tests because normal data is a basic assumption in parametric testing. Normality test was conducted, and values of skewness and kurtosis (acceptable not more than +3 & not less than -3) are presented in Table 2. These values show that the normality assumption is met so that we can proceed with multiple regression analysis. Moreover, the VIF values (acceptable VIF < 10) also suggest that there are no issues with multicollinearity in the collected data.

### 4.1 Multiple regression analysis

Multiple regresson analysis using the stepwise method was performed to determine the impact of independent variables on project performance. The results are presented in Tables 4 and 5. The stepwise method adds independent variables one by one in the model to determine impact on the outcome. Table 4 shows that R^2^ of model 1 was .242, which means 24.2% variation in project performance was due to independent variable employee involvement. In model 2, R^2^ was .270, which means a 27% variation in project performance was due to employee involvement and operation focus. Model 3 shows that the value of R^2^ was .280, meaning that 28% variance occurred in project performance due to three independent variables: employee involvement, operation focus, and commitment. After comparison, Model 3 came out to be a better model based upon the higher R^2^ value.

**Table 4:** Multiple regression results summary.

Model	R	R Square	Adj R Square	F Change	Sig.
1	.492^a^	.242	.240	103.68	.000^a^
2	.520^b^	.270	.266	12.41	.000^b^
**3**	**.529** ^c^	**.280**	**.273**	**4.14**	**.043** ^c^

Note: Dependent Variable is project performance for all 3 models presented in table 2 above. Model 3 is best fit out of 3 models.

^a^ Predictors: (Constant), Commitment.

^b^ Predictors: (Constant), Commitment, Operation focus.

^c^ Predictors: (Constant), Commitment, Operation focus, Employee involvement.

**Table 5:** Coefficients of multiple regression model.

Model 3		B	SE B	β	t	Sig.
Predictors	Commitment	.417	.099	.300	4.219	.000
	Operation focus	.223	.096	.142	2.324	.021
	Employee Involvement	.231	.114	.159	2.036	.043

Multiple regression model coefficients are presented in Table 5. R-square for model 3 was 28%, with an adj R-square was 27.3%. The multiple regression model revealed that employee involvement (β = .159, p< .05), operation focus (β = .142, p < .05) and commitment (β = .300, p< .05) all had a significant positive impact on project performance. Consequently, H1, H2, and H6 were accepted. The results also revealed that H3, H4, H5, and H7 were not supported.

### 4.2 Mediation analysis

Mediation analysis was performed to assess the mediating role of commitment on the association between employee involvement and project performance. The results provided in Table 6 revealed that the total effect of employee involvement on project performance was significant (β = .686, p < .05). The direct impact of employee involvement on project performance was also significant (β = .345, p < .05). The indirect effect of employee involvement on project performance through commitment was found significant (β = .341, p < .05). This shows that the association amongst employee involvement and project performance is mediated by management commitment. Hence, hypothesis H8 was supported.

**Table 6:** Mediation test summary.

Total Effect		Direct Effect		Indirect Effect			
Coefficient	p	Coefficient	p	Coefficient	SE	LLCI	ULCI
.686	.000	.345	.001	.341	.077	.177	.478

## 5. Discussion

This study finds that commitment, which is one of the total quality management principles has demonstrated a significant positive effect on the performance of a construction project. This shows that the top management commitment has strong potential to affect the performance of a project in the construction industry of Pakistan. This research outcome is consistent with a few studies where it was found that senior management and leaders guiding the quality system and assessing the financial and non-financial activities resulted in better organizational performance, quality performance and safety programs [63, 64]. The firm’s success in applying the best quality management methods depends on how seriously top management takes the deployment of a quality environment. If senior management is not committed, it is impossible to apply quality management standards across the firm [65]. The top leadership uses quality systems to establish organizational standards and enhances staff engagement to achieve better quality goals and success for the firm [66]. Our study also illustrates the importance of employee involvement as it is significantly associated with project performance in the Pakistan construction industry. Involved and committed employees understand customer needs and make efforts to address them. This finding is consistent with earlier research where employee involvement, encouragement and participation were found to be essential workforce practices for continuous improvement [67, 68]. Another fundamental principle of workforce involvement relates to employee performance management, which significantly impacts the project’s performance [69, 70]. Employee training, participation, and involvement were found to be essential elements of operational performance [71]. The success of ongoing improvement is directly correlated with employee engagement and dedication [72, 73]. On the other hand, the construction industry relies heavily on its employees during the construction process, and employee involvement is positively correlated to project performance. This demonstrates that employee involvement in the construction industry of Pakistan is essential to influence project performance.

Clear goals and objectives fixed by companies with clearly defined methods will eventually lead teams to improved performance. The current study has verified this assertion, where the key performance index (KPI) was found to be an essential process management element for the project’s success [20]. Another important element of process management relates to operations which significantly impact the project’s performance [5, 51]. Many other studies have confirmed that process and operation management strongly impact overall performance [74]. Sadikoglu & Olcay [71] discovered that customer service, inventory and innovation performance all contributed to organizational performance in Turkey’s industrial and service industry. Zeng et al. [51] explored the impact of management systems on manufacturing performance in 8 different countries and observed that operation/process management was found to impact performance significantly. Regardless of region, process and operation have shown their significance in enhancing performance. The outcomes of these studies highlight the significance of implementing a well-defined process over the project’s entire life cycle. Another important finding of this study is that the linkage between employee involvement and project performance is mediated by management commitment. The current study illustrates the importance of employee involvement as it is significantly associated with project performance in the Pakistan construction industry through the mediating role of management commitment. Employee participation and involvement in organizational functions were found to have a significant effect on senior management commitment. Interestingly, commitment is concerned with employees’ involvement and participation for better organizational performance [56]. Another study has shown a strong relationship between employee involvement and management commitment via organizational support [75]. A greater level of employee participation and personal commitment to organizational success is established with the support and dedication of top management [76].

Other results of this research show that customer focus, measurement, analysis and knowledge management, continuous improvement, and strategic planning have an insignificant impact on project performance in the local construction industry. Client/Customer focus has an insignificant link with project performance. This specifies that fulfilling customer satisfaction and understanding the needs of the clients/customers do not take priority in the construction sector of Pakistan. Similar surprising findings appeared in previous researchs where consumer satisfaction and retention including focus on the client/customer were not considered essential TQM practices for organizational performance [77, 78]. Our study also finds that client/customer focus is insignificant in enhancing project performance and it is not surprising because it shows Pakistan’s construction sector dominated by mostly personal and family owned businesses still does not consider the significance of putting customer needs first.

This study also finds that strategic planning has an insignificant effect on the performance of a project in construction firms. This finding is consistent with earlier research where planning was not found to be an essential TQM principle for better quality performance [77]. Zwikael et al. [41] examined the association between planning and success with moderating effect of risk and discovered an insignificant relationship between planning, project effectiveness and efficiency. They also discovered the moderating role of risk; high risk increased the planning requirements and low risk increased the effectiveness of the project. Planning is considered a significant factor for project success in project management and strategic management literature. In the construction sector, some practices such as reliance on owners, designers, and contractors, consistently fluctuating project objectives and goals, and engagement of numerous experts make strategic planning more difficult [5]. While in the context of construction management, planning a project may be difficult, especially for construction projects. This is due to the fact that construction and building projects are notoriously intricate, dynamic, unusual, risky and unpredictable [79–81]. It is hard to forecast the results of construction projects due to these characteristics. During the early phases of a project, construction managers often pay little attention to detailed or strategic planning [82]. Client-related problems such as uncertainty in client requirements, frequent fluctuations in project scope and a lack of solid work breakdown structures (WBSs) to support clients’ frequent change orders all contribute to this [83, 84]. Subsequently, project planning is not found significant to affect the performance of a project in Pakistan’s construction industry.

Measurement, analysis, and knowledge management are very important elements of TQM. This research shows measurement, analysis & knowledge management have an insignificant impact on project performance. This finding is unexpected and inconsistent with few studies. Mehralian et al. [47] argued availability of quality-related data and usage was considered one of the total quality management elements that significantly influence the performance of Iran’s pharmaceutical industry. Ooi et al. [44] discovered a negative relationship between information analysis and innovation performance. Similarly, Teh et al. [85] discovered a negative relationship between information analysis and the automotive industry of ASEAN. Our observation of lack of support for measurement, analysis & knowledge management in enhancing project performance is not surprising because it shows Pakistan’s construction sector does not consider the significance of using data and information in decision-making and does not allocate resources for this essential activity.

It is also found that continuous improvement does not significantly affect the performance of construction projects which is also inconsistent with other studies. Arief et al. [86] argued a significant impact of continuous improvement on the financial performance of the manufacturing sector. Jørgensen et al. [87] conducted a study to examine the relationship between CI, HRM and the effect of CI on performance. The authors discovered a significant effect of CI on performance. Generally, these studies showed that increased continuous improvement activities improve the overall organizational performance. Unfortunately, in the current study, CI is insignificant in enhancing project performance because it shows Pakistan’s construction sector still does not consider the significance of continual improvement in products and services.

Commitment, operation focus, and employee involvement were recognized as the most important pillars of TQM in construction projects. Unfortunately, some key principles of TQM, i.e., customer focus, continuous improvement, strategic planning, measurement, analysis and knowledge management were not recognized as the most important pillars in the construction industry of Pakistan. As per the report [88], the highly sensitive problems and risks in the construction industry of Pakistan are uncertainty in financial management, fluctuation in material prices, natural disaster, delays, and poor designs. All of these risks do not have any connection with the principles of total quality management in the construction industry of Pakistan. Moreover, comparing the findings of this research with other developing countries specifically on project-based organizations [5, 89], it became evident that while each country’s culture, characteristics, and other aspects are distinct, these developing nations also emphasized on employee involvement, operations-related activities, and management support as in Pakistan. However, they do not adhere to the culture of focusing on consumer feedback, project planning, measurement, analysis, and ongoing improvement. They have simply not taken these crucial factors into account like developed and industrialized nations.

## 6. Conclusion

The importance and implementation of TQM specifically in construction industry has emerged as a key factor towards delivering successful projects besides contributing to improved organizational performance and project success. The construction sector is viewed as a significant contributor to the Pakistan economy. This study revealed that operation focus, employee involvement, and management commitment significantly affect project performance in the Pakistan construction industry. Employee involvement also has a significant positive impact on the project’s performance through the mediating role of management commitment. The current TQM principles show that employee involvement is important in the construction industry of Pakistan. Employee involvement is the main component that retains the operations of the construction project, and every project phase depends on employee involvement. Furthermore, operations and process-related activities are significant in this unpredictable, dynamic, and unique industry. Well-defined operations and processes enhance productivity. The participation of top-level managers, supervisors, and owners in quality-related activities and functions enhances the performance of a project. Other TQM principles such as customer focus, strategic planning, continuous improvement and measurement, analysis & knowledge management had insignificant impact on construction project performance and needs further research.

### 6.1 Practical implication

This study demonstrated the partial effect of TQM on project performance. The outcomes revealed that only three out of seven total quality management elements significantly correlated with construction project performance. Therefore, it is understandable for owners, managers and supervisors that the implementation of total quality management elements (commitment, employee involvement, and operation focus) enhanced project performance. The implication is that construction firms should focus on those TQM practices, including commitment, employee involvement, and operation focus, to improve the performance of construction projects. Construction companies need to invest in resources in order to collect, measure and analyze data related to customer satisfaction and project performance in order to ensure continuous improvement in their services. This study also guides the policy decision makers to encourage construction firms to embark on a total quality management system, enhance their organizational and project performance, and improve the economy.

### 6.2 Limitations and future directions

The study had few limitations because of time constraints and resources. The first constraint was that the research only concentrated on companies in Pakistan. It is recommended that research should be expanded to different developed and developing countries. Secondly, the research focuses only on the construction industry and in future may target different industries including software, manufacturing and service industry. Another future research direction is to perform qualitative research on TQM factors which were found insignificant in the local industry and determine their role in achieving project success. Moreover, top management commitment could also be tested as a moderator between TQM initiatives and project success.

## Supporting information

S1 Appendix Measurement scales. (DOCX)
