# Correction: Exploring the impact of total quality management initiatives on construction industry projects in Pakistan

**DOI:** 10.1371/journal.pone.0326896

**Published:** 2025-06-23

**Authors:** Nimra Afzal, Aamer Hanif, Muhammad Rafique

Reference 7 in this article [1] was retracted before publication. This reference is replaced with [2].

The Data Availability statement is updated to: All relevant data are available from the authors on request.
