# Supplementary material for: Exploring the impact of total quality management initiatives on construction industry projects in Pakistan
Source: PLoS One. 2022 Sep 27;17(9):e0274827. doi: 10.1371/journal.pone.0274827 (PMC9514645; doi:10.1371/journal.pone.0274827)
Supplement: S1 Appendix — (DOCX) [file pone.0274827.s001.docx]

Appendix

**Commitment**

|  | Organization’s top management (senior executives, major department heads, and supervisors) views improvement in quality as a way to increase profits. |
| --- | --- |
|  | Organization’s top management has objectives for quality performance. |
|  | Organization’s top management is evaluated for quality performance |
|  | Organization’s top management assumes responsibility for quality performance |
|  | Organization’s top management provides personal leadership for quality products and quality improvement |
|  | Major department heads within the organization participate in the quality improvement process |
|  | Quality issues are reviewed in organization’s management meetings. |
|  | Communication links are established between employees and top management. |

**Employee Involvement**

|  | Training in advanced statistical techniques is given to the employees who need training. |
| --- | --- |
|  | The organization forms teams to solve problems. |
|  | Employees possess sufficient knowledge of the basic aspects of the organization’s sector. |
|  | Employees understand the basic processes used to make organization’s products/services. |
|  | Managers and supervisors participate in specialist training. |
|  | Resources are available for employee quality training in the organization. |
|  | Employees are recognized for superior quality improvement |
|  | Hourly/non-supervisory employees are involved in quality decisions |
|  | The organization gives feedback to employees on their quality performance. |
|  | The organization’s management adapt to the new ideas that employees come up with. |

**Customer Focus**

|  | The organization frequently is in close contact with its customers. |
| --- | --- |
|  | The organization actively and regularly seeks customer inputs to identify their needs and expectations. |
|  | The organization informs customers’ current and future needs and expectations to its employees effectively. |
|  | The organization’s customers give feedback on quality and delivery performance. |
|  | Customer complaints are used as input to improve the organization’s processes. |
|  | The organization measures customer satisfaction systematically and regularly. |
|  | Employees know who the organization’s customers are. |

**Continuous Improvement**

|  | The organization has a quality improvement program. |
| --- | --- |
|  | The organization practices continuous improvement in all of its products, services and processes. |
|  | The management ensures that employees are capable of taking initiatives and assimilating better ways of doing their jobs. |
|  | The organization designs processes to be “mistake-proof” to minimize the changes of errors. |
|  | The organization makes extensive use of statistical techniques to reduce variation in processes. |
|  | The organization gives clear, comprehensive, and standardized documentation about work methods and process instructions to employees. |
|  | Continuous improvement processes are based on a systematic assessment of organizational effectiveness |
|  | Benchmarking techniques are used to establish improvement standards and objectives. |
|  | Self-assessment processes take place on a regular basis. |

**Strategic Planning**

|  | Our organization has a mission statement which has been communicated throughout the company and is supported by our employees. |
| --- | --- |
|  | Our organization has a comprehensive structured planning process which regularly sets and reviews short and long-term goals. |
|  | Our organizations always incorporates supplier capabilities and needs of other stakeholders including the community when we develop our plans, policies and objectives. |
|  | Our organization strategic plans and tactical plan are linked to quality values. |
|  | Our organization integrates continuous quality improvements into planning process. |

**Operation Focus**

|  | Our organization develops a set of key work processes. |
| --- | --- |
|  | Our organization establishes Key Performance Indicators (KPIs) for monitoring purpose. |
|  | Our organization practices daily operation work processes report system. |
|  | Our organization regularly monitors and reviews on work processes performance. |
|  | Our organization uses of approaches or tools to improve process performance and reduce variability. |
|  | Our organization exercises two-way communication with suppliers. |
|  | Our organization has a well-prepared disaster and emergency preparedness system to ensure the continuity of our operations. |

**Measurement, analysis & knowledge management**

|  | Decisions are usually made at the level where the best information is available. |
| --- | --- |
|  | Working in this organization is like being part of a team |
|  | My organization continuous invests in the skills of employees |
|  | There is a clear and consistent set of values in this organization that governs the way we do business |
|  | When disagreements occur, we work hard to achieve solutions  that benefit both parties in the disagreement |
|  | People from different organizational units still share a common perspective |
|  | This organization is very responsive and changes easily |
|  | Customer input directly influences our decisions |
|  | We view failure as an opportunity for learning and improvement |
|  | My organization has a clear mission that gives meaning and direction to our work. |
| 1. : | The leadership has clearly stated the objectives we are trying to meet |
|  | We have a shared vision of what this organization will be like in the future |

**Project Performance**

|  | The project was successful in terms of quality of the project outcome |
| --- | --- |
|  | The project was successful in terms of scope and requirements of the project being met |
|  | The project was successful in terms of timeliness of project completion. |
|  | The project was successful in terms of costs and efforts being under budget or within estimates. |
